# Dr. Gaylord Parsons Clark

**Published:** 1907-10

**Authors:** 


Dr. Gaylord Parsons Clark, Dean of the College of Syracuse University and Professor of Physiology in Medicine, died at his home in Syracuse, September 1, 1907. On the day after registration at the opening of the college, a meeting of students and faculty was held, at which recognition of Dean Clark's long and efficient service and of his noble character was made by the Chancellor and others.

